# A Sensitive Membrane-Targeted Biosensor for Monitoring Changes in Intracellular Chloride in Neuronal Processes

**DOI:** 10.1371/journal.pone.0035373

**Published:** 2012-04-10

**Authors:** Spencer D. Watts, Katherine L. Suchland, Susan G. Amara, Susan L. Ingram

**Affiliations:** 1 Department of Neurobiology, Medical School, University of Pittsburgh, Pittsburgh, Pennsylvania, United States of America; 2 Department of Neurological Surgery, Oregon Health & Science University, Portland, Oregon, United States of America; Yale School of Medicine, United States of America

## Abstract

**Background:**

Regulation of chloride gradients is a major mechanism by which excitability is regulated in neurons. Disruption of these gradients is implicated in various diseases, including cystic fibrosis, neuropathic pain and epilepsy. Relatively few studies have addressed chloride regulation in neuronal processes because probes capable of detecting changes in small compartments over a physiological range are limited.

**Methodology/Principal Findings:**

In this study, a palmitoylation sequence was added to a variant of the yellow fluorescent protein previously described as a sensitive chloride indicator (YFPQS) to target the protein to the plasma membrane (mbYFPQS) of cultured midbrain neurons. The reporter partitions to the cytoplasmic face of the cellular membranes, including the plasma membrane throughout the neurons and fluorescence is stable over 30–40 min of repeated excitation showing less than 10% decrease in mbYFPQS fluorescence compared to baseline. The mbYFPQS has similar chloride sensitivity (k_50_ =  41 mM) but has a shifted pKa compared to the unpalmitoylated YFPQS variant (cytYFPQS) that remains in the cytoplasm when expressed in midbrain neurons. Changes in mbYFPQS fluorescence were induced by the GABA_A_ agonist muscimol and were similar in the soma and processes of the midbrain neurons. Amphetamine also increased mbYFPQS fluorescence in a subpopulation of cultured midbrain neurons that was reversed by the selective dopamine transporter (DAT) inhibitor, GBR12909, indicating that mbYFPQS is sensitive enough to detect endogenous DAT activity in midbrain dopamine (DA) neurons.

**Conclusions/Significance:**

The mbYFPQS biosensor is a sensitive tool to study modulation of intracellular chloride levels in neuronal processes and is particularly advantageous for simultaneous whole-cell patch clamp and live-cell imaging experiments.

## Introduction

GABA and glycine are the main inhibitory neurotransmitters in the nervous system. These neurotransmitters activate ionotropic receptors that flux chloride to produce inhibition of many cell types. However, in some cells, activation of these receptors produce excitatory effects through various mechanisms, including differences in chloride/bicarbonate permeability of GABA_A_ channels [1], [2], [3], differences in chloride gradients [4], [5], [6] and altered intracellular chloride homeostasis [7], [8], [9], [10], [11], [12], as well as synaptic integration mechanisms [2], [3], [13], [14]. Depolarizing GABA_A_ responses are crucial in early development for establishing and maintaining synaptic connections in neurons throughout the brain [15], [16] and become hyperpolarizing after the induction of chloride cotransporter subtype 2 (KCC2) expression during postnatal development [11], [17], [18]. Modulation of chloride co-transporter function has also been implicated in nervous system disorders, including temporal lobe epilepsy [19] and neuropathic pain [20], [21]. Clearly, regulation of intracellular chloride is becoming recognized as an important neuronal process in synaptic plasticity of neuronal circuits.

Many of the neurotransmitter transporters, such as the dopamine transporter (DAT), norepinephrine transporter and excitatory amino acid transporters have been demonstrated to elicit uncoupled chloride conductances in the presence of substrates [22], [23], [24], [25]. Very little is known about the physiological role of these chloride conductances. Although the DAT-mediated chloride current has been shown to increase firing of DA neurons [25], it has been difficult to study this current using whole-cell patch-clamp recordings from the soma when DAT proteins are localized to small neuronal processes [26]. Thus, development of a sensitive fluorescent biosensor for chloride that could be used for monitoring changes in chloride in processes of neurons would significantly enhance information obtained with simultaneous whole-cell patch-clamp experiments.

Fluorescence imaging of cellular events provides a noninvasive window into cellular function not available with other approaches. Fluorescent tools include both synthetic dyes and the GFP (AvGFP) derived fluorescent proteins used as both cellular markers and biosensors. Synthetic chloride-sensitive dyes include 6-methoxy-*N*-(3-sulfopropyl)quinolinium (SPQ), 6-methoxy-*N*-ethyquinolinium chloride (MEQ) and *N*-(ethoxycarbonylmethyl)-6-methoxyquinolinium bromide (MQAE). These dyes have been used to monitor intracellular chloride in neurons *in vitro* [2], [10], [27], [28]. The fluorescence emission of yellow fluorescent protein (YFP), a derivative of GFP, has been shown to respond rapidly and reversibly to changes in the concentration of small anions such as Cl^−^ and has been used as a genetically encoded biosensor for chloride [29], [30], [31]. Clomeleon, a ratiometric chloride biosensor, is a chimeric protein combining the chloride-sensitive YFP with the chloride-insensitive cyan-fluorescent protein (CFP) and uses fluorescence resonance energy transfer (FRET) to monitor intracellular chloride concentrations in neurons and transgenic animals [31], [6]. Point mutations in the YFP gene have been discovered that dramatically increase the affinity of YFP to chloride [30], [32] and have been used to monitor the chloride conductance of cystic fibrosis transmembrane conductance regulator (CFTR) [33] and for high-throughput fluorescence screening of CFTR active compounds [34]. These mutations have also been incorporated into a chimera analogous to Clomeleon [31], [35].

Genetically encoded biosensors have advantages when compared to currently available chloride-sensitive dyes. These advantages include excitation at visible wavelengths that avoid issues associated with phototoxicity caused by excitation wavelengths near the UV range, good signal-to-noise ratios at low concentrations, and insensitivity to gluconate and other physiological anions [32], [35]. Other advantages arise from the fact that these biosensors can be specifically targeted to subcellular compartments, including the plasma membrane with addition of genetically encoded subcellular localization signal peptides.

The goal of this study was to demonstrate that attaching a palmitoylation site to the YFP variant with enhanced sensitivity to chloride developed by Galietta and colleagues (YFP-H148Q/V163S or YFPQS [32]), targets the YFPQS protein to the plasma membrane (mbYFPQS) and that this provides enhanced functionality of the protein. Anchoring the biosensor to the membrane decreases leakage or diffusion of the fluorescent protein into the recording pipette during whole-cell recordings. *In situ* calibration of mbYFPQS expressed in midbrain neurons indicates that this construct has enhanced sensitivity to low chloride concentrations and is less influenced by changes in cellular pH compared to YFP and the FRET-based biosensor Clomeleon. These results confirm the increased chloride sensitivity of YFPQS developed by Galietta and colleagues and demonstrate the usefulness of this particular variant for detecting changes in intracellular chloride concentrations in neurons. In addition, the results show that mbYFPQS is suitable for monitoring changes in intracellular chloride concentrations in both the soma and processes of midbrain neurons in response to the GABA_A_ agonist muscimol and DAT substrates.

## Results

### Properties of mbYFPQS as a Chloride Sensor

**Figure 1:**
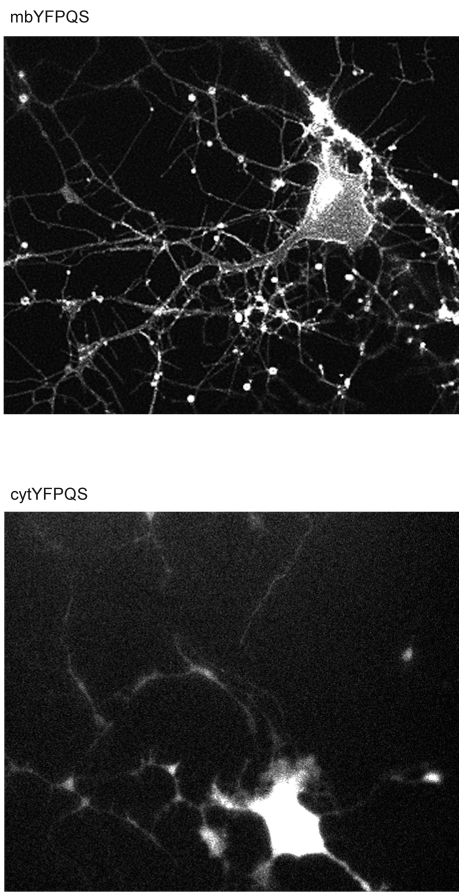
Fluorescence images of cultured midbrain neurons expressing either mbYFPQS or cytYFPQS. Single Z-stack images (1 µm) at mid-soma level taken 3 days post-transfection. Note that the fluorescence of the processes of the cytYFPQS-expressing cell is difficult to observe without saturating the fluorescence of the soma.

In this study, the chloride sensor described by Galietta and colleagues, YFP-H148Q/V163S (YFPQS [32]) has been further modified by the addition of the N-terminal signal peptide of Neuromodulin. The addition of this peptide results in post-translational palmitoylation of the protein, which facilitates the anchoring of the protein to the cytoplasmic face of membranes, including the plasma membrane [36]. Figure 1 shows a comparison of the cytoplasmic construct (cytYFPQS) and the membrane-targeted construct (mbYFPQS) expressed in midbrain neurons.

**Figure 2:**
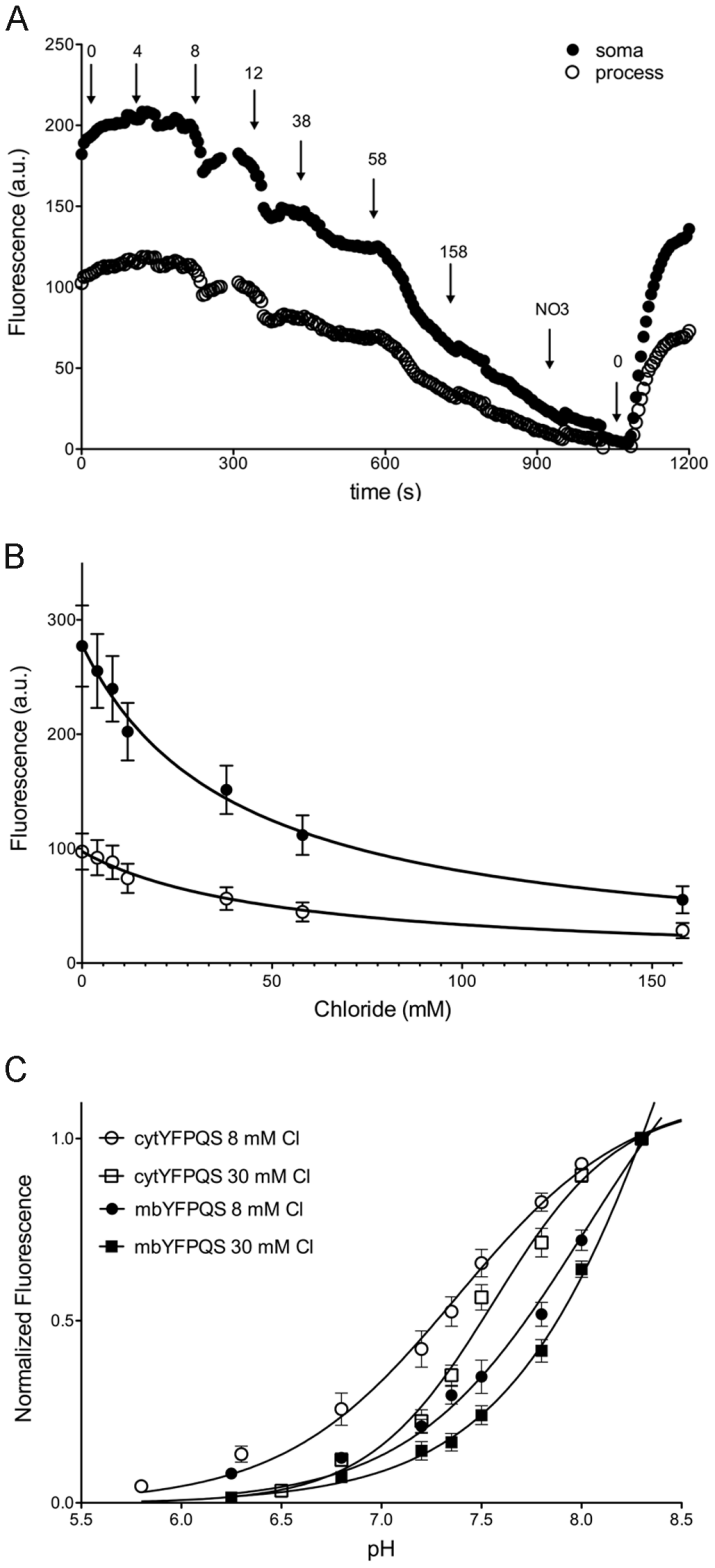
Chloride and pH calibration of mbYFPQS expressed in midbrain neurons. Cultured midbrain neurons expressing mbYFPQS were incubated with the following ionophores for 20–30 min, tributyltin-Cl (1 µM), nigericin (1 µM) and valinomycin (1 µM) prior to superfusion of a high potassium extracellular solution containing different final chloride concentrations. A. Plot of fluorescence versus chloride concentration for the soma and a process in a representative cell. A solution substituting nitrate (NO_3_) for chloride was use to quench the mbYFPQS fluorescence at 158 mM NO_3_ to verify that measured fluorescence was due to mbYFPQS. Concentrations of chloride are denoted by numbers and arrows. Responses are averages of 4–6 points just prior to next chloride concentration. B. The mbYFPQS fluorescence was plotted versus chloride concentration for the soma (n  =  18) and processes (n  =  9). Data were fit with the equation Y  =  F0/(1 + ([Cl^−^_int_]/k_50_)). There was no significant difference in k_50_ (F(1, 10)  =  1.710, p > 0.05). R square values for fits were 0.993 and 0.985 for the soma and process, respectively. C. pH calibration of mbYFPQS and cytYFPQS expressed in midbrain neurons. Following incubation of neurons in ionophores as in A & B, neurons were exposed to high potassium extracellular solutions containing either 8 mM or 30 mM Cl^−^ at various pH (n  =  5–8/group). Fluorescence of mbYFPQS was normalized to the fluorescence at pH 8.3 and plotted versus pH. Data were fit with sigmoidal curves to estimate apparent pK_a_.

To determine the sensitivity range of mbYFPQS to changes in intracellular chloride, midbrain cultures were perfused with an extracellular solution of varying chloride concentrations in the presence of ionophores to equilibrate extracellular and intracellular chloride concentrations as described (see Methods) [37]. The fluorescence of mbYFPQS was inversely dependent upon the concentration of chloride in the extracellular solution, with significantly reduced fluorescence at the highest chloride concentration (158 mM Cl^−^) and greatest fluorescence in the absence of extracellular chloride (0 mM Cl^−^). This is depicted in a plot of steady state fluorescence as a function of chloride concentration (Figure 2A). The average k_50_ for all cells imaged was 41 ± 3 mM (n  =  17) and did not vary substantially from the k_50_ measured from distal processes of 52 ± 6 mM. The non-palmitoylated cytYFPQS had a k_50_  =  59 ± 18 mM (n  =  8) when expressed in cultured midbrain neurons, comparable to both mbYFPQS and purified YFPQS [32].

### pH Sensitivity of mbYFPQS

A potential complication with YFP-based assays is that YFP fluorescence emission is also affected by pH because the anion-sensing mechanism of YFP involves a shift in chromophore pK_a_ [29], [30]. To determine the contribution that pH fluctuations might have on intracellular chloride measurements using this sensor, mbYFPQS was expressed in midbrain neurons and the intracellular pH was adjusted by equilibration with ionophore-containing buffers of varying pH in either 8 mM or 30 mM chloride (Figure 2C). These pH concentrations cover the estimated intracellular chloride concentrations within midbrain neurons [38]. The apparent pK_a_s for mbYFPQS were 8.0 ± 0.2 and 8.5 ± 0.2 at 8 mM and 30 mM Cl^−^, respectively. These apparent pK_a_s were not significantly changed when permeabilizing the cells with β-escin (80 µM [39]) instead of ionophores with pK_a_s  =  7.8 ± 0.1 and 8.4 ± 0.4 at 8 mM and 30 mM Cl^−^, respectively. The cytYFPQS had apparent pK_a_s of 7.4 ± 0.1 and 7.6 ± 0.1 at 8 mM and 30 mM Cl^−^, respectively when expressed in midbrain neurons, similar to results determined for purified YFPQS (pK_a_  =  7.23 [32] and the apparent pK_a_ of 7.3–7.8 for the new CFP-YFP ratiometric chloride indicator over the chloride concentration range of 10–60 mM [35]). Thus, the pKa of mbYFPQS is shifted to the right compared to the cytoplasmic constructs and only small differences in the fluorescence intensity were observed over a typical intracellular pH range (pH 7.5 to 7.2; Figure 2C).

### Loss of Fluorescence during Whole-cell Patch-clamp Recordings

**Figure 3:**
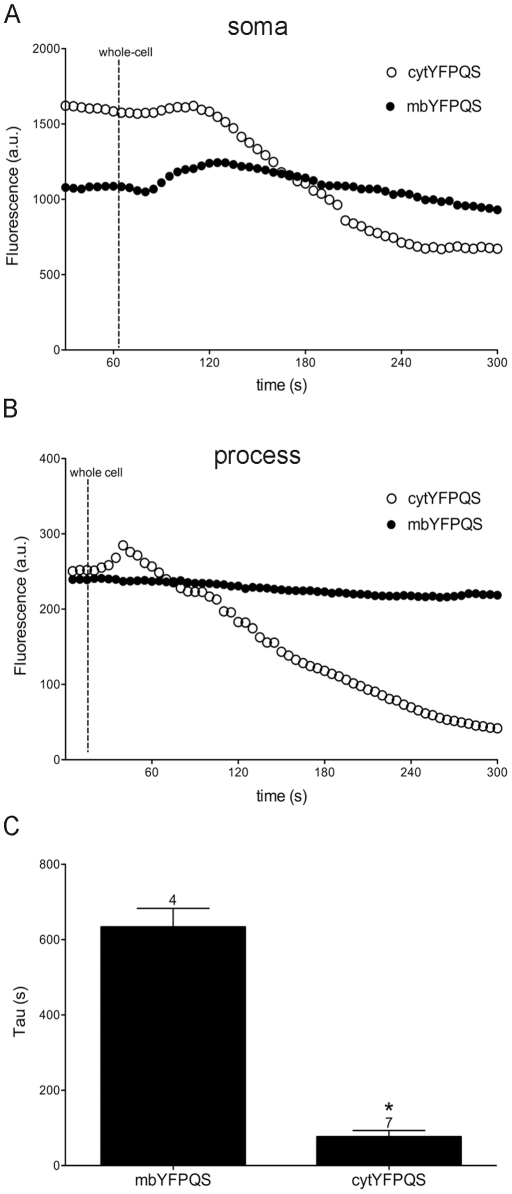
mbYFPQS stabilizes fluorescence decay during whole-cell patch-clamp recordings. A & B. Examples of fluorescence decay following whole-cell patch-clamp recordings from cultured midbrain neurons expressing mbYFPQS or cytYFPQS showing that fluorescence decreases faster for the cytYFPQS-expressing cells. C. In order to determine the rate in more homogeneous cells, whole-cell patch-clamp recordings with potassium methylsulfate pipette solution from HEK293 cells expressing mbYFPQS or cytYFPQS were compared. Bar graph in C compares the fluorescence decay tau (t-test, t(9)  =  13.01, *p < 0.05).

One advantage of the membrane-targeted construct is that it will not readily diffuse during whole-cell patch-clamp recordings. In order to test this directly, whole-cell recordings from HEK-293 cells expressing either cytYFPQS or mbYFPQS were made and the fluorescence intensity (200 ms exposure at 0.2 Hz) was monitored over time. The tau of fluorescence decay in recordings from cytYFPQS-expressing cells (76 ± 17 s, n  =  7) was significantly faster than when recording from mbYFPPQS-expressing cells (633 ± 50 s, n  =  4; t(9)  =  13.01, p < 0.01; Figure 3). The faster decay suggests that the intracellular pipette solution dialyzes the cytYFPQS more readily than the mbYFPQS.

### Detection of Changes in Intracellular Chloride with Activation of GABA_A_ Receptors in Midbrain Neurons

**Figure 4:**
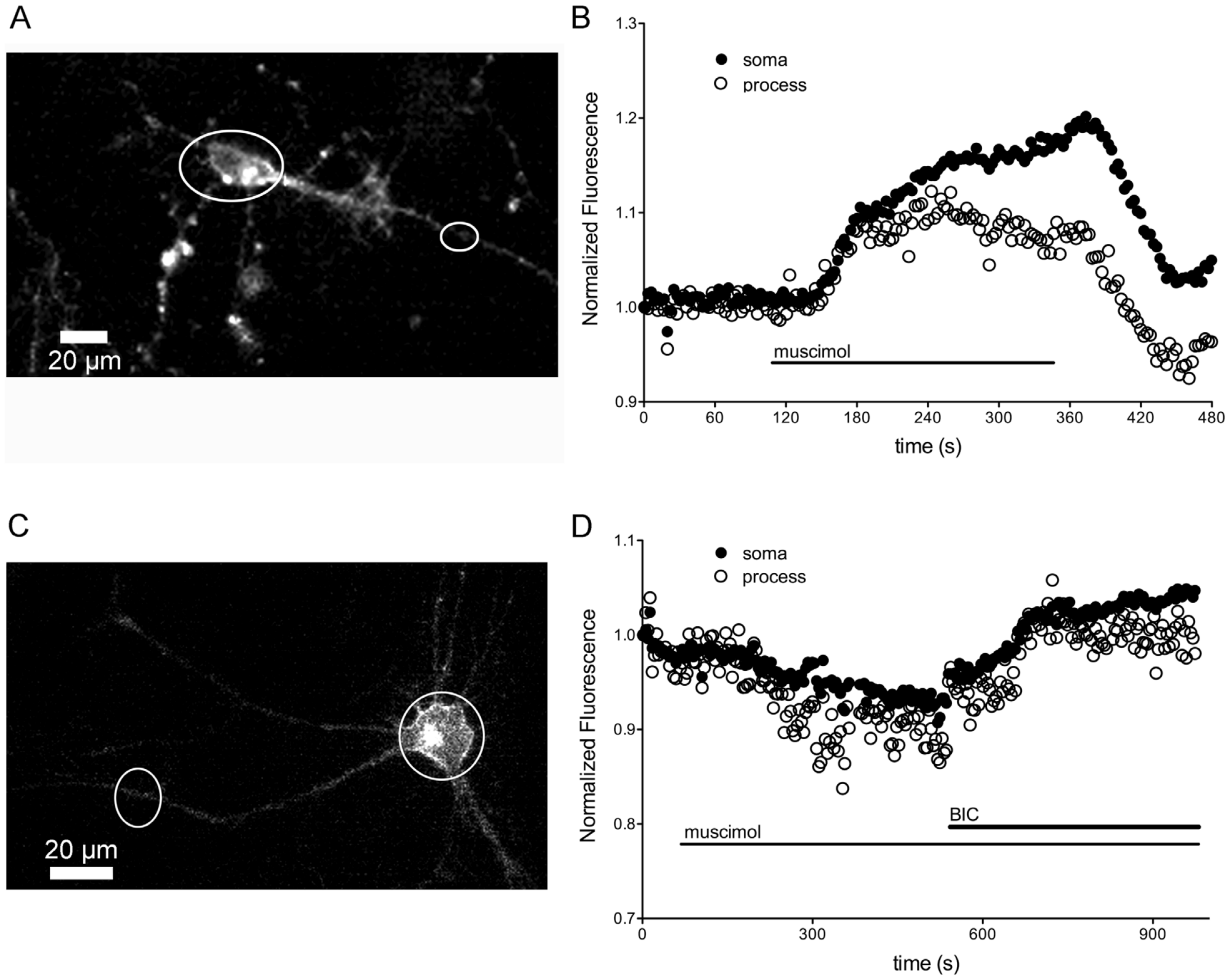
Muscimol-induced changes in mbYFPQS fluorescence illustrate that mbYFPQS can detect both inwardly- and outwardly-directed chloride gradients in different cell populations. A. Fluorescence image of cultured midbrain neuron expressing mbYFPQS. B. Increase in fluorescence induced by the GABA_A_ agonist, muscimol (20 µM) in the soma (closed circles) and process (open circles) of cell depicted in A. Fluorescence was normalized to initial baseline fluorescence. C & D. Decrease in fluorescence induced by the GABA_A_ agonist, muscimol (20 µM) in the soma (closed circles) and processes (open circles) of cell depicted in *C*. The decrease in fluorescence is reversed with superfusion of the GABA_A_ antagonist bicuculline (10 µM). No consistent differences were observed with regions of interest drawn at different distances from the soma.

In order to test the utility of mbYFPQS as a tool to monitor changes in intracellular chloride concentrations, we activated the GABA_A_-mediated chloride flux with bath application of muscimol (20 µM; Figure 4) in the presence of physiological extracellular solution (158 mM Cl^−^). Muscimol superfusion of neurons expressing mbYFPQS resulted in both fluorescence increases and decreases highlighting the ability of mbYFPQS to detect fluorescence changes in both directions. In the majority of the cultured neurons (18/20), muscimol superfusion resulted in increased mbYFPQS fluorescence (Figure 4A & B) suggesting that the chloride flux is outward, as expected from cultures of neurons taken from developmentally immature P2–P4 rats [11]. In contrast, the fluorescence decrease observed in a small subpopulation of cells is indicative of inward chloride flux, perhaps indicating a maturing cellular phenotype or a different subpopulation of neurons (Figure 4C & D). The muscimol-mediated fluorescence changes were inhibited by superfusion of the GABA_A_ antagonist, bicuculline (10 µM) (Figure 4D). In addition, the absolute value of the change in mbYFPQS fluorescence induced by muscimol in cells and processes of the midbrain neurons were comparable demonstrating that there is no substantial difference in the sensitivity of the sensor when expressed in these two regions. The muscimol-induced change in fluorescence was significantly reduced and/or reversed by the chloride exchange inhibitor bumetanide (25 µM; data not shown) suggesting that active transport of chloride establishes the chloride gradients observed in the neurons.

### Detection of Amphetamine-mediated Changes in Intracellular Chloride

**Figure 5:**
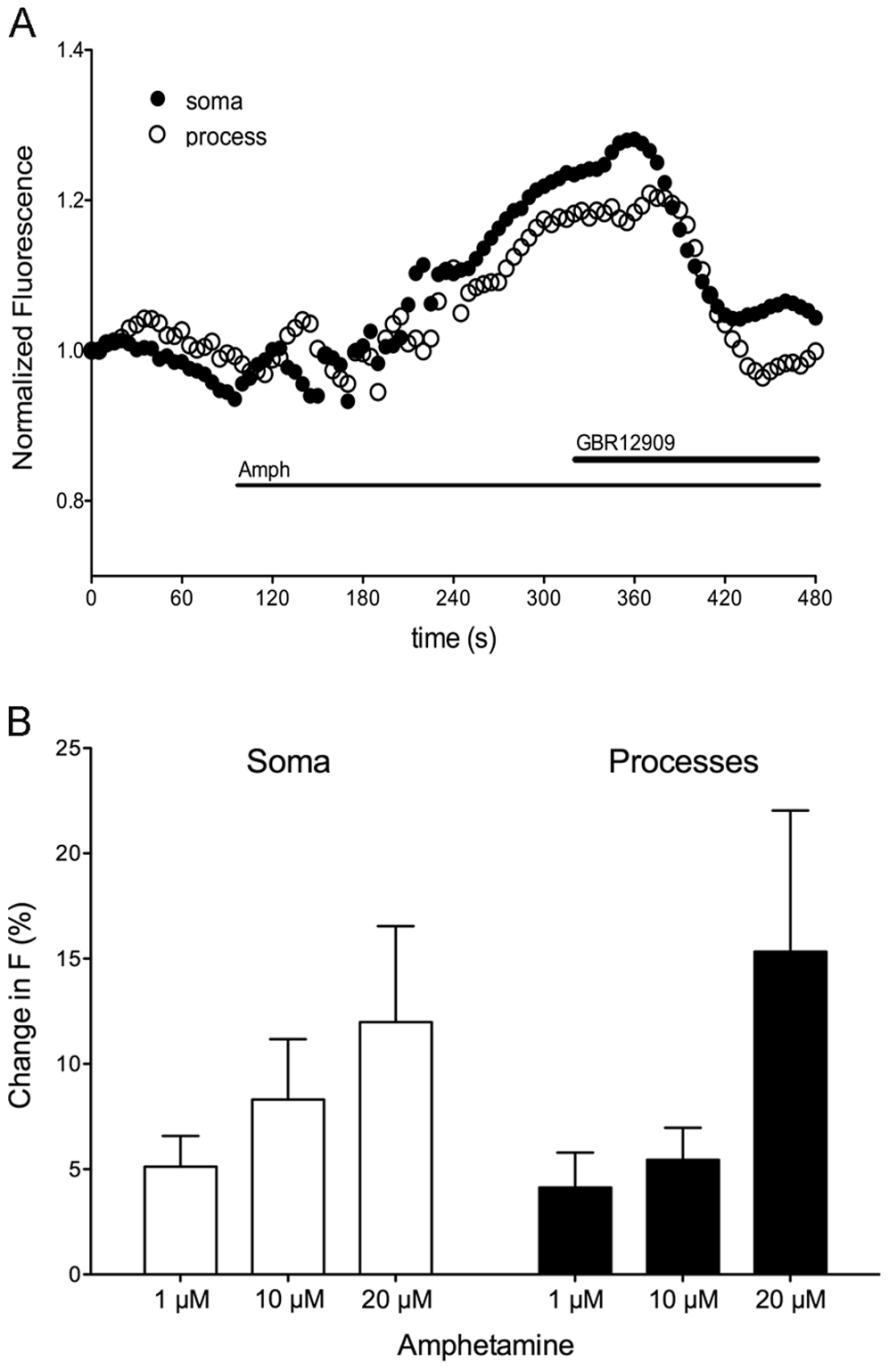
Amphetamine-induced changes in mbYFPQS fluorescence. A. Amphetamine increased the fluorescence of both the soma (closed circles) and process (open circles) of a cultured midbrain DA neuron expressing mbYFPQS. The selective DAT inhibitor, GBR12909 (10 µM) reversed the change in fluorescence. B. Bar graph showing dose-dependent fluorescence changes mediated by amphetamine in both soma and processes of cultured midbrain neurons. N  =  7 (1 µM), 3 (10 µM) and 3 (20 µM).

We previously demonstrated that DAT substrates stimulate an uncoupled chloride current associated with the DAT in midbrain DA neurons [25]. Since DAT are expressed in axons and dendrites of DA neurons, we were interested in determining if chloride changes to DAT substrates could be detected with mbYFPQS. Amphetamine (1–20 µM) superfusion elicited increases in mbYFPQS fluorescence in a subpopulation of the cultured midbrain neurons (Figure 5A) that were reversed by the DAT-specific inhibitor, GBR129009 (10 µM). The change in mbYFPQS fluorescence elicited by amphetamine was dose-dependent in both soma and processes of the responding neurons (Figure 5B).

### Stability of mbYFPQS for Simultaneous Imaging and Whole-cell Patch-clamp Recordings

**Figure 6:**
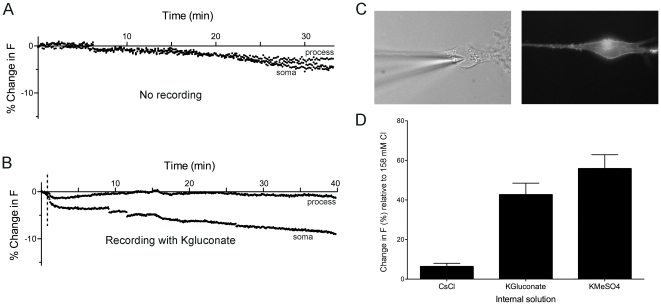
Minimal photobleaching of mbYFPQS occurs with repeated photo-stimulation. A. A cultured midbrain neuron expressing mbYFPQS was imaged with an exposure of 100 ms every 5 s for 35 min with less than 6% decrease in baseline fluorescence. B. A neuron imaged with the same parameters (100 ms exposure, every 5 s) that was whole-cell patch-clamped with potassium gluconate internal solution (12.6 mM Cl^−^). A decrease in fluorescence was observed with break-in to whole-cell mode (denoted by dotted line) but after reaching a new stable baseline, there was less than 5% decrease in fluorescence over 35 min of imaging. C. Brightfield and epifluorescence image of cell imaged in *B*. D. Bar graph of % increase in fluorescence of typical internal solutions (CsCl (134 mM Cl^−^); KGluconate (121 mM Gluconate/12.6 mM Cl^−^); KMeSO4 (121 MeSO4/12.6 mM Cl^−^)) compared to fluorescence in high intracellular chloride concentrations (158 mM Cl^−^). Experiments were done in perforated mbYFPQS-expressing neurons after incubation in ionophores used for chloride and pH calibration experiments. The results show that gluconate and methylsulfate ions do not appreciably quench the mbYFPQS fluorescence.

In order to determine if mbYFPQS will be useful for simultaneous imaging and whole-cell patch-clamp experiments, cultured midbrain neurons were transfected with mbYFPQS and control experiments tested the photobleaching by repeated exposure to light excitation wavelengths for 30–40 min (Figure 6A). Less than 10 ± 5% (n  =  5) decrease in baseline fluorescence occurred when imaging at a rate of 0.2 Hz and that rate of photobleaching was not significantly different during recordings with potassium gluconate internal solution (8 ± 4% decrease; n  =  4; Figure 6B).

The YFPQS construct has previously been shown to be differentially sensitive to different anions [32]. The DAT-mediated chloride current is also sensitive to anion substitution in the intracellular recording solutions [25]. For example, the DAT-mediated current is blocked by intracellular substitution with gluconate but methylsulfate ions have a similar permeability to chloride ions [25]. However, it is not known how these intracellular anions interact with the fluorophore in mbYFPQS. In order to test the sensitivity of mbYFPQS to these anions, cells were incubated in the cocktail of ionophores used for chloride calibration experiments (see Figure 2) but the extracellular solution was replaced with our typical internal solutions (pH  =  7.35). The fluorescence of mbYFPQS in the presence of these solutions was compared to the quenching observed with the normal extracellular solution (158 mM Cl^−^) after ionophore perforation of the membrane (Figure 6D). The CsCl internal solution (134.2 mM Cl^−^) slightly increased the fluorescence by 6 ± 2% (n  =  22). Potassium gluconate internal solution (12.6 mM Cl^−^) increased fluorescence by 43 ± 6% (n  =  17) and potassium methylsulfate substitution (12.6 mM Cl^−^) increased fluorescence by 56 ± 7% (n  =  17). The increase in fluorescence with potassium methylsulfate substitution is the expected increase from calibration experiments shown in Figure 2 with a shift in chloride concentrations from 132 mM to 12 mM. These results indicate that the gluconate and methylsulfate ions have little effect on mbYFPQS fluorescence and will be useful anion substitutions for whole-cell patch-clamp experiments.

## Discussion

The fluorescence emission of yellow fluorescent protein (YFP) has been shown to respond rapidly and reversibly to changes in the concentration of small anions such as Cl^−^ and has been used as a genetically encoded biosensor for chloride [30], [40]. The results in this paper demonstrate that the point mutations in YFP described by Galietta and colleagues [32] increase the chloride sensitivity and are useful for monitoring changes in intracellular chloride. Intracellular chloride concentrations have been estimated to be 5–17 mM in the soma of midbrain neurons [38] but concentration gradients of >20 mM between soma and dendrites also have been observed in some neurons [6] suggesting that intracellular chloride gradients from 5–30 mM may be observed. We have further adapted the YFP variant (YFPQS) by addition of a palmitoylation sequence (mbYFPQS) that facilitates anchoring of the protein to the plasma membrane and have demonstrated that this construct has increased sensitivity to chloride (k_50_ ∼40 mM) after expression in cultured midbrain neurons compared to the parent YFP molecule (k_50_ =  168 mM) and the FRET-based CFP-YFP chimera, Clomeleon (k_50_  =  160 mM [6], [31]). This increased sensitivity is similar to the cytYFPQS [32] and a variant with a triple mutation in YFP (k_50_ ∼30 mM; [35] and also is comparable to the best chloride dye that is commercially available (MQAE; k_50_  =  2–40 mM [10], [28]). In addition, the mbYFPQS has a shifted pKa compared to the cytYFPQS and the FRET-based sensors [35]. Finally, we demonstrate that the membrane targeting is advantageous for stabilizing the fluorophore during whole-cell patch-clamp recordings.

The advantages of a genetically encoded chloride sensor are significant when compared to conventional chloride-sensitive dyes, such as the quinoline-based fluorescent dyes SPQ, MEQ and MQAE. First, mbYFPQS can be directed to different compartments of the cell by generating a genetically encoded fusion protein with the biosensor and specific subcellular localization sequences. In this case, the mbYFPQS construct encodes a signal peptide from neuromodulin, which results in post-translational palmitoylation of the protein that facilitates attachment to the cytoplasmic face of cellular membranes. This is a particular advantage when opting for simultaneous fluorescence imaging and whole-cell patch-clamp electrophysiological experiments because the mbYFPQS does not diffuse into the pipette or leak out of cells similarly to cytoplasmic constructs or dyes. Although the quinoline-based dyes have good sensitivity in cells (SPQ k_50_  =  ∼80 mM [27]; MEQ  =  ∼50 mM [11], [41]; MQAE  =  2–40 mM [10], [28]), insensitivity to pH and relatively low biological toxicity, they are problematic during long experiments because of fluorescence reduction due to leaking across the membrane and bleaching of the fluorophore. Thus, the most successful experiments monitoring changes in chloride with these dyes have been done using 2-photon microscopy [28], [42]. On the other hand, the fluorescence of mbYFPQS is very stable over time compared to the chloride-sensitive dyes, MQAE [10] and MEQ [41] and the ratiometric FRET-based Clomeleon [6]. It is bright, very sensitive and the fact that the fluorescence excitation is within the visible light range limits cellular damage during experiments. The bright fluorescence also allows short exposure times (100–300 ms), a contributing factor to the minimal bleaching of the fluorescence during experiments using conventional epifluorescence wide-field microscopy.

The main disadvantage of all of the YFP-based chloride sensors is that the fluorophore is also sensitive to changes in pH in a near physiological range. Purified YFPQS has a pKa of 7.27 at 0 mM Cl^–^ that increases to 7.97 at 75 mM Cl^–^ [32]. Clomeleon has a pKa range of 5.2 (0 mM Cl^−^) to 6.5 (150 mM Cl^−^) [31] and the triple mutation biosensor has a pKa of 7.1 to 8.0 over the same range [35]. Calibration of mbYFPQS expressed in midbrain neurons determined apparent pK_a_s of 8.0 and 8.5 at 8 mM and 30 mM Cl^−^, respectively. These pKas are shifted to the right compared to the cytYFPQS expressed in midbrain neurons and purified YFPQS [32], as well as to Clomeleon-based chloride sensors [31], [35] suggesting that small physiologically relevant pH changes in the neurons will not significantly affect mbYFPQS fluorescence. We determined that direct measurements of changes in pH in the cultured midbrain neurons due to muscimol-induced GABA_A_ currents were negligible using a GFP-based pH sensor (data not shown). In actuality, intracellular pH is tightly regulated [43] and may not contribute greatly to the fluorescence changes observed for mbYFPQS. However, it is possible that pathological processes may cause larger pH changes so direct measurements with selective pH sensors should be considered for each application.

In addition to the important role in development, alterations in chloride homeostasis have been shown to be important in regulating neuronal network activity in adults (for review, see [44]). Adult olfactory sensory neurons [28] and photoreceptors [45], [46] have higher intracellular chloride concentrations than many cell types and typically display depolarizing effects of GABA_A_ and glycine agonists. Even more interesting is the demonstration in several areas that GABA_A_ responses can be modulated by transcriptional or translational regulation of chloride co-transporters. Some cells have a chloride gradient between the soma and axons [31] and/or dendrites [6], [47], while the neurons in the suprachiasmatic nucleus can shift GABA responses on a circadian cycle [48]. Other studies have also shown that synaptic activity is sufficient to alter intracellular chloride concentrations [49] or membrane voltage to result in depolarizing responses to GABA [50]. Finally, disruption of chloride homeostasis due to downregulation of KCC2 may play an important role in abnormal neuronal activity associated with temporal lobe epilepsy [51] and neuropathic pain [52]. These studies suggest that there are multiple mechanisms by which neuronal activity is modulated by intracellular chloride.

Few studies have looked at chloride homeostasis in midbrain neurons even though GABA neurotransmission regulates activity of both dopaminergic and GABAergic neurons in the substantia nigra (SN) and ventral tegmental area (VTA) of the midbrain [38], [53]. GABAergic neurons of the SN have been shown to be more sensitive to GABA_A_-mediated inhibition compared to dopaminergic neurons [54], [55] because the GABAergic neurons express KCC2 whereas dopaminergic neurons do not [38]. These results suggest that adult dopaminergic neurons regulate chloride homeostasis differently than other midbrain neurons. This data is consistent with observations that the chloride equilibrium potential for GABA_A_ receptor activation was more hyperpolarized than for the chloride current mediated by the dopamine transporter in processes of midbrain dopamine neurons [25]. Further studies will use expression of mbYFPQS in midbrain dopamine neurons to facilitate monitoring of intracellular chloride levels associated with DAT activity in neuronal processes combined with simultaneous whole-cell patch-clamping to study chloride homeostasis and endogenous DAT-mediated signaling.

## Materials and Methods

### Ethics Statement

Experiments were conducted in accordance with the animal care and use guidelines outlined in the Guide for the Care and Use of Laboratory Animals and approved by respective Institutional Animal Care and Use Committees (Washington State University ASAF#3711 and OHSU#1192).

### Generation of Chloride Biosensor

The YFP with enhanced sensitivity to low chloride concentrations (YFPQS) was generated from pEYFP-mem (Clontech), which encodes the N-terminal palmitoylation signal sequence of neuromodulin fused to EYFP, by the following amino acid substitutions in the EYFP protein, H148Q (CAC → CAG) and V163S (GTG → AGC). The mutagenesis was verified by fully sequencing the EYFP gene in plasmid p-mbYFPQS.

### Cell Culture and Transfection

Ventral mesencephalic cells including dopamine neurons from substantia nigra and ventral tegmental area were cultured as described [56]. Briefly, 2–6 day old Sprague-Dawley rat pups were anesthetized with halothane (Sigma-Aldrich). Ventral midbrains were dissected and incubated in a dissociation medium (in mM): 116 NaCl; 5.4 KCl; 26 NaHCO_3_; 25 glucose; 2 NaH_2_PO_4_; 1 MgSO_4_; 1.3 cysteine 0.5 EDTA; 0.5 kynurenate containing 20 units/ml papain, at 34–36°C under continuous oxygenation for two hours. The tissue was triturated with fire polished Pasteur pipettes in *glial medium* (minimum essential medium with 10% heat-inactivated fetal bovine serum, 0.45% D-glucose, 5 pg/ml insulin, 0.5 mM glutamine, penicillin, and streptomycin). Dissociated cells were pelleted by centrifugation at 500 × g for 10 minutes, resuspended and plated on poly-L-lysine (100 µg/ml) and laminin (50 µg/ml) coated glass coverslips. One hour after plating, the medium was changed to *neuronal medium* (50% minimum essential medium, 39% hams-F12 medium, 10% heat-inactivated horse serum, 1% heat-inactivated fetal bovine serum, 0.45% D-glucose, 5 pg/ml insulin and 0.1 mg/ml apotransferrin). Cortical glia were cultured in the presence of *glial medium* in 75 cm^2^ tissue culture flasks coated with poly-D-lysine and laminin. *Neuronal medium* was conditioned over these glia overnight, supplemented with 1ng/ml GDNF and 500µM kynurenate and filter sterilized before feeding to mesencephalic cultures. Cultures were transfected 7–10 days post plating for 1–2 hours with 3 µl Lipofectamine 2000/1 mg DNA in 300 ml MEM per coverslip or with 0.8 µl NeuroMag/4 nmoles DNA (NeuroMag Magnetofection, Oz Biosciences). Cells were imaged 1–4 days after transfection.

Human Embryonic Kidney 293 (HEK293) cells were maintained in Advanced DMEM media (Gibco) supplemented with 10% fetal bovine serum and 100 µg/mL streptomycin and 100 U/mL penicillin at 37°C and 5% CO_2_ in a humidified atmosphere. 24 hours prior to transfection, cells were trypsinized and plated on 12 mm coverslips coated in poly-L-lysine (BD Biosciences). For transfection, each coverslip of cells was incubated with 0.25 µg of plasmid and 1.75 µl of NeuroMag (NeuroMag Magnetofection, Oz Biosciences) for 20 minutes. Cells were imaged 24–48 hours post-transfection.

### Imaging

Measurements of mbYFPQS fluorescence were performed by placing a coverslip of cultured cells in a continuous perfusion chamber on an inverted microscope equipped with a PixelFly cooled CCD camera, a Sutter High Speed Filterwheel and software control via InCyte acquisition software (Intracellular Imaging, Inc.) or a set-up comprising an upright AxioExaminer, AxioCam MRm and Axiovision software for data acquisition (Carl Zeiss, Inc.) equipped with a Xenon (Intracellular Imaging, Inc.) or Mercury lamp (XCite 120Q, Lumen Dynamics Group) and filter sets for YFP fluorescence (Excitation 500/dichroic VIS >500–600/Emission 545; Omega Optical). Cells were bath perfused in normal extracellular buffer containing in mM: 146 NaCl, 5 KCl, 5 HEPES, 2.5 CaCl_2_ and 1.2 MgCl_2_ at pH 7.35 and room temperature.

### Calibration of Chloride Dependence

For chloride calibration experiments, a high potassium (100 mM) extracellular solution with potassium chloride substituted for sodium chloride and containing the ionophores tributyltin-Cl (1–10 µM, Sigma-Aldrich), nigericin (1–10 µM, Sigma-Aldrich) and valinomycin (1 µM, Sigma-Aldrich) were used [37]. Equimolar substitutions of potassium gluconate for potassium chloride were made for final chloride concentrations of 158, 58, 38, 12, 8, 4 and 0 mM. Nitrate substitutions were used to fully quench fluorescence in calibration experiments. In a separate set of experiments, ß-escin (80 µM, Sigma-Aldrich) was used to permeabilize cells [39]. Fluorescence intensity versus chloride concentration was plotted and fit with the equation Y  =  F0/(1 + ([Cl^−^_int_]/k_50_)) [37].

### Calibration of pH Dependence

For pH calibration, the high potassium solution with added ionophores was made at final chloride concentrations of 8 mM and 30 mM and varying pH. The fluorescence was normalized to fluorescence intensity at pH 8.3 and calibration curves were fit with a sigmoidal curve to estimate apparent pKa [35] for mbYFPQS under the two different chloride concentrations.

### Determination of Whole-cell Patch-clamp-induced Quenching of mbYFPQS

The YFPQS construct has previously been shown to be differentially sensitive to different anions [32]. Since the whole-cell patch-clamp recording technique introduces the pipette solution to the intracellular environment of the recorded neuron, three typical internal solutions were compared for their ability to quench mbYFPQS fluorescence. A cesium chloride internal solution (total chloride  =  134.2 mM) contained in mM: 130 CsCl, 10 HEPES, 1.1 EGTA, 2 MgCl_2_ and 0.1 CaCl_2_, pH 7.35. The potassium gluconate solution (total chloride  =  12.6 mM) contained in mM: 138 KGluconate, 10 HEPES, 1 EGTA, 10 KCl, 1 MgCl_2_ and 0.3 CaCl_2_, pH 7.35 and the potassium methylsulfate solution (total chloride  =  12.6 mM) substituted potassium methylsulfate for the potassium gluconate.

### Data Analyses

Average pixel intensity in each region of interest (ROI) was normalized to the baseline fluorescence intensity of each ROI by subtracting the fluorescence of a background ROI in the field and dividing by the initial fluorescence of each ROI. Percent (%) change in fluorescence was determined after background subtraction by [(fluorescence in drug)-(fluorescence in control)]/(fluorescence in control) * 100%. Calibration data were plotted and curve fits to equations (above) were used to obtain values of k_50_ and pKa (GraphPad Prism). All results are expressed as mean ± STE and ANOVA with *post-hoc* tests when appropriate (GraphPad Prism).
